# A Case of Femoral Neck Insufficiency Fracture due to Tumor-Induced Osteomalacia

**DOI:** 10.1155/2021/6668006

**Published:** 2021-10-06

**Authors:** Yu Inoue, Tomoaki Fukui, Keisuke Oe, Shinya Hayashi, Teruya Kawamoto, Ryosuke Kuroda, Takahiro Niikura

**Affiliations:** Department of Orthopaedic Surgery, Kobe University Graduate School of Medicine, 7-5-1, Kusunoki-cho, Chuo-ku, Kobe 650-0017, Japan

## Abstract

Tumor-induced osteomalacia (TIO) is a rare skeletal disease caused by hypersecretion of fibroblast growth factor 23 (FGF-23) from neoplasms of mesenchymal origin; patients with TIO present with insufficiency fractures, progressive bone pain, and delayed fracture unions. Herein, we report the case of a 48-year-old man with an insufficiency fracture in his left femoral neck associated with TIO. The causative tumor located in the patient's maxillary sinus had been resected; however, complete resection was impossible due to the location of the tumor. Therefore, the patient's osteomalacia persisted, and he experienced a left femoral neck fracture in the absence of severe trauma. Because delayed fracture union was anticipated in this patient, we performed an internal fixation using an implant with a lateral plate for angular stability and multiple screws for rotational stability. Although fracture union took 15 months, the patient's postoperative course was uneventful, and he could walk without any symptoms or assistance at his most recent follow-up 30 months after surgery. In TIO, hypersecretion of FGF-23 leads to increased renal excretion of phosphorus, increased bone resorption of calcium and phosphorus, decreased osteoblastic bone mineralization, and decreased gastrointestinal absorption of calcium and phosphorus, leading to insufficiency fractures and delayed fracture unions. Diagnosis of TIO is often delayed due to its rarity and vague symptoms. Total resection of the causative tumor is the optimal treatment; however, in cases wherein complete tumor resection is impossible, drug therapy may be insufficient, and the underlying TIO pathology, including bone fragility, may persist. Early diagnosis of TIO is important for preventing insufficiency fractures; however, when fractures are unavoidable, the surgical treatment of femoral neck fractures in patients with osteomalacia should account for a longer time frame for complete fracture union and therefore utilize implants with sufficient stability.

## 1. Introduction

Tumor-induced osteomalacia (TIO) is a skeletal disease induced by the inhibition of phosphorus reabsorption in the proximal renal tubules due to the action of fibroblast growth factor 23 (FGF-23) produced by a tumor [1, 2]. The main symptoms of TIO include easy fracture, joint pain, muscle weakness, and gait disturbance [1]. The most common causative tumor type is a phosphaturic mesenchymal tumor (mixed connective tissue variant), which accounts for 70–80% of all cases of TIO [3]. Complete resection of the tumor can lead to rapid improvement and resolution of all symptoms. However, if the tumor remains unresected or partially resected, persistent osteomalacia could lead to delayed fracture unions due to disordered bone calcification [4]. Herein, we report a case of insufficiency fracture of the left femoral neck due to TIO resulting from a partially resected tumor.

## 2. Case Presentation

This case involved a 48-year-old man whose chief complaint upon his first visit to our department in 2016 was left hip pain.

**Figure 1:**
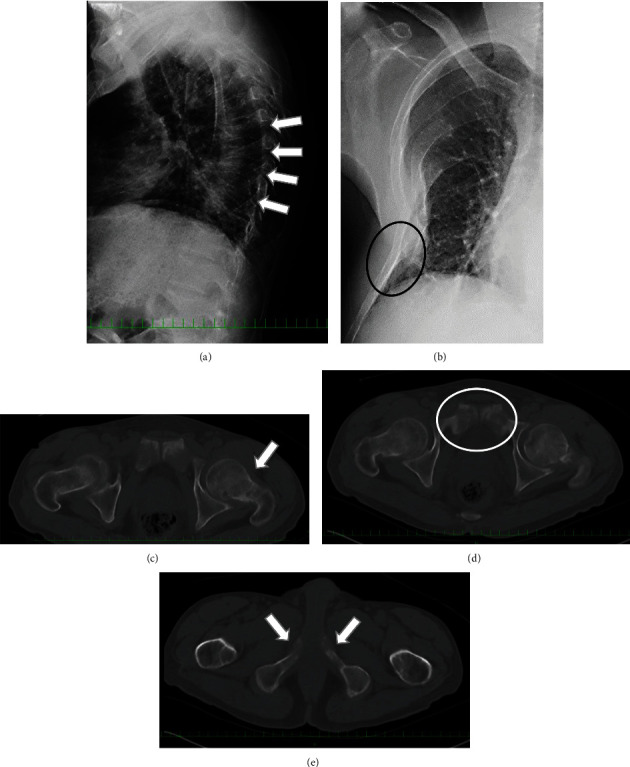
Radiographs taken in 2007 showing multiple vertebral fractures (a, white arrows) and right rib malunion (b, black circle). A left femoral neck insufficiency fracture (c, white arrow) and deformity of both pubes (d, white circle) and ischia (e, white arrows) are shown in computed tomography images in 2007.

In 2006, the patient visited a nearby hospital for lower back pain and was noted to have multiple compression fractures in his lumbar spine on radiography and an old rib fracture on computed tomography (CT). Blood tests at that time showed elevated alkaline phosphatase (864 IU/L), low phosphorus (1.4 mEq/L), and normal calcium (9.9 mEq/L) levels; therefore, in 2007, he was referred to the endocrinology department in our hospital. After extensive examination, multiple vertebral compression fractures, right rib fractures, a left femoral neck insufficiency fracture, deformity of both pubes and ischia (Figure 1), short stature, and systemic bone atrophy were noted. Further examinations revealed impaired phosphorus absorption and a vitamin D activation disorder that showed a high intact parathyroid hormone (PTH) (76 pg/mL) level. Consequently, TIO was suspected at that time; however, a causative tumor could not be located, which is relatively common in cases of TIO. Therefore, the patient was treated with oral calcium and phosphorus.

**Figure 2:**
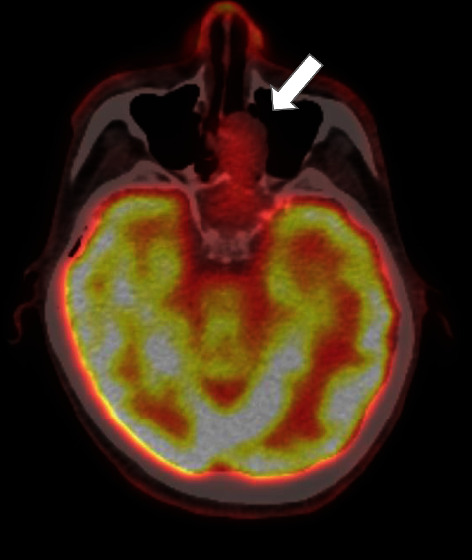
A neoplastic lesion (white arrow) is shown in the maxillary sinus on a positron emission tomography scan in 2013.

In 2012, a neoplastic lesion was observed in the maxillary sinus on positron emission tomography (PET) (Figure 2) and considered the causative tumor. Initially, the nasal cavity of the patient was subjected to biopsy. The pathologists stained the specimen for FGF-23; however, a negative result was obtained, which was consistent with phosphaturic mesenchymal tumor in the nasal cavity and paranasal sinus. Based on imaging findings combined with pathological reports and other examination results, the patient was diagnosed with TIO. Otolaryngologists attempted to surgically excise the tumor; although total resection was not possible due to its location, resection of most of it was performed. The patient's serum FGF-23 level, which was more than 800 pg/mL before tumor resection, decreased to 741 pg/mL after the surgery. However, it gradually increased to 3700 pg/mL at 45 months postoperatively. Furthermore, his 1,25-dihydroxyvitamin D3 level was normal at 56 pg/mL in 2016, and intact PTH level, which was initially as high as 76 pg/mL in 2013, gradually increased to 85 pg/mL in 2016.

**Figure 3:**
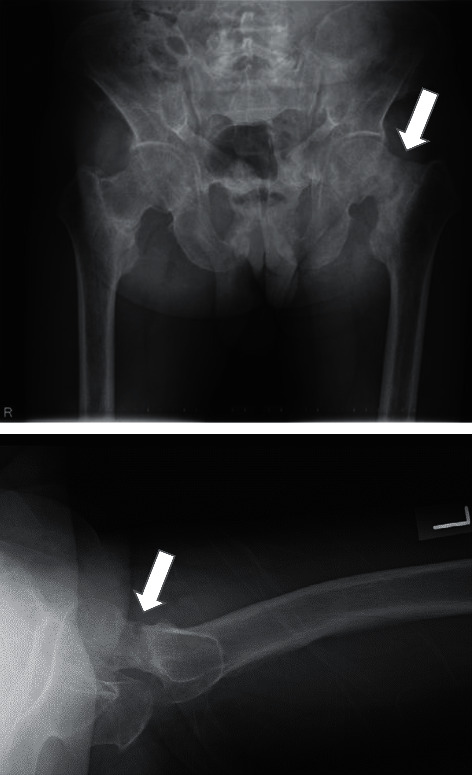
Radiographs in 2016 revealing left femoral neck fracture and varus deformity at the fracture site.

**Figure 4:**
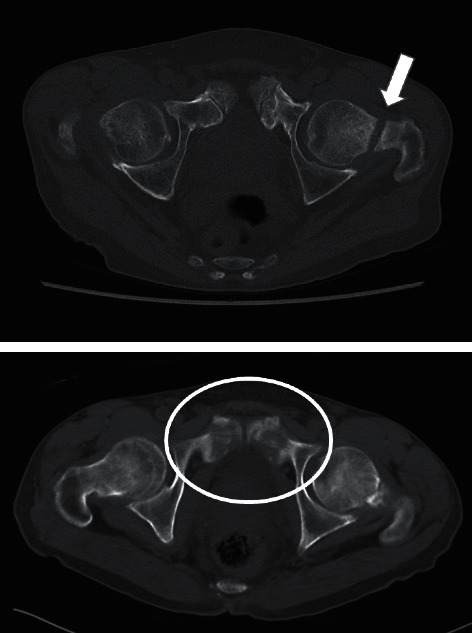
Axial view of the hip joint via computed tomography in 2016 revealing a left femoral neck fracture (white arrow). It revealed an old fracture of the pubic bone (white circle).

**Figure 5:**
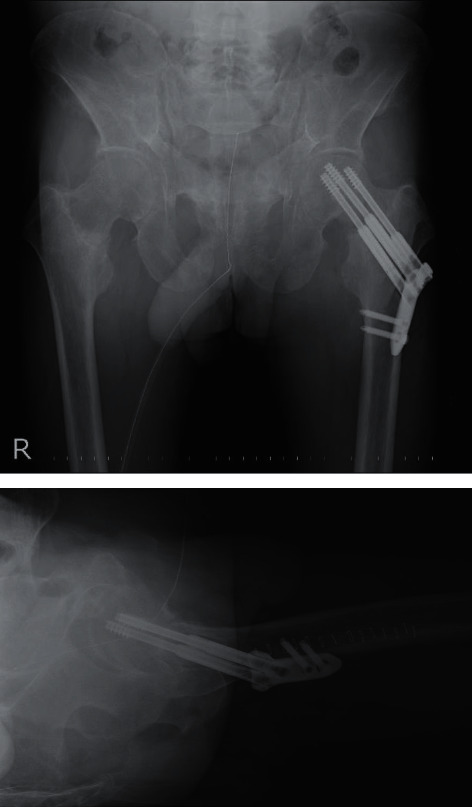
Radiographs taken immediately after surgery. The fracture was fixed using an implant with multiple compression hip screws and a side plate.

**Figure 6:**
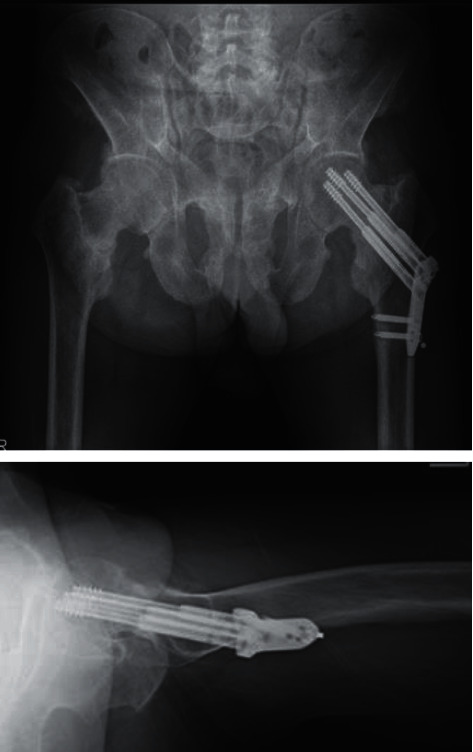
Radiographs taken 15 months after surgery revealing that the fracture achieved bone union.

Subsequently, he experienced a sudden onset of mild left hip pain. Thereafter, upon descending a flight of stairs, the patient's left hip pain increased, and he visited the endocrinology department in our hospital and was referred to our department. On physical examination, he could stand and walk independently; however, the pain was induced by both internal and external rotations of his left hip joint. The range of motion in both hip joints was restricted to 90° of flexion and 20° of abduction in the left hip joint and 110° of flexion and 30° of abduction in the right hip joint. A left femoral neck complete fracture was observed on radiography (Figure 3) and CT (Figure 4). Osteosynthesis was performed using TresLock® (KiSCO Co., Ltd., Kobe, Japan), an implant with multiple compression hip screws and a side plate (Figure 5). Bone samples that were collected around the fracture site during the surgery were submitted for pathological examination. Many fragments of the cortex bone were observed, and there was no finding of osteomalacia. Partial weight-bearing on the left leg was allowed immediately after surgery. Low-intensity pulsed ultrasound was initiated 3 months after surgery, and callus formation was gradually observed. Full weight-bearing was permitted 15 months after surgery upon the confirmation of fracture union (Figure 6). Although the patient's postoperative course was uneventful, a long period was required for bony union to occur. At the most recent follow-up 30 months postoperatively, the patient could walk independently without any symptoms.

## 3. Discussion

TIO is a rare disease in which hypersecretion of FGF-23 produced by a tumor leads to the inhibition of phosphorus reabsorption in the proximal tubules, increased bone resorption of calcium and phosphorus, suppressed osteoblastic bone mineralization, and decreased gastrointestinal absorption of calcium and phosphorus, resulting in bone fragility [1, 2]. The combined effect of these pathologic mechanisms induces a state of osteomalacia, with symptoms presenting in the form of gradual progressive bone pain, myopathies, fractures, and generalized muscle weakness [3]. Previous studies have reported that it often takes many years to identify the causative tumor after the onset of symptoms because of their rare occurrence and vague symptoms [5, 6].

The majority of TIO-induced femoral neck insufficiency fractures can be treated conservatively with rest and medication if the symptoms, including hip pain, are not severe [7, 8]. However, patients with progressively worsening fracture displacement should be treated surgically [8, 9]. In a report on surgical treatments for a femoral neck fracture due to TIO, bipolar head arthroplasty led to early loosening due to bone fragility [10]. In another report, total hip arthroplasty following corrective osteotomy was applied to a chronic phase case after a femoral neck fracture due to TIO because the fracture site was too severely deformed to be treated by conventional internal fixation [11]. Another report of femoral neck fracture due to osteomalacia described that even if the initial fracture reduction was acceptable, progressive varus deformity of the femoral neck could occur postoperatively, leading to vertical shearing forces across the fracture site that could contribute to nonunion [12]. Furthermore, because osteomalacia results in a reduction in the compression strength of the bone, it was reported that refracture occurred after internal fixation in four of six cases of subtrochanteric fractures [13]. On the contrary, there has been one report on a case of valgus osteotomy that achieved bone union [14]. Based on these studies, we propose that rigid osteosynthesis should maintain fracture stability considering the bone fragility and delayed union associated with TIO.

In the current case, although the diagnosis of TIO had been previously confirmed, the tumor could not be completely surgically resected, and the effects of medication were limited, which resulted in an insufficiency fracture due to bone fragility. Therefore, we determined that rigid osteosynthesis was required in this case, and we used an implant with a side plate for angular stability and multiple screws for rotational stability. We suspected that the use of an implant with these characteristics ultimately led to a successful bone union without failure of internal fixation; however, bony fusion did take 15 months to occur.

Delayed diagnosis of TIO can lead to weakness of the bony architecture due to prolonged inhibition of bone matrix mineralization and resultant insufficiency fractures. For early diagnosis, monitoring serum alkaline phosphatase and phosphate levels as well as screening for muscle weakness, joint pain, and gait disturbance is crucial and can prevent additional serious complications. When insufficiency fractures are detected in young people, clinicians should consider the possibility of bone metabolism disorders, including osteomalacia and, if necessary, make an early referral to an endocrinologist.

In conclusion, we successfully completed the surgical treatment of a femoral neck insufficiency fracture in a patient with TIO whose causative tumor could not be completely removed. Based on our experience with this case, we strongly believe that rigid osteosynthesis and careful postoperative follow-up are important for achieving successful outcomes in such cases.
